# Cartilage diversification and modularity drove the evolution of the ancestral vertebrate head skeleton

**DOI:** 10.1186/s13227-023-00211-1

**Published:** 2023-05-05

**Authors:** Zachary D. Root, David Jandzik, Claire Gould, Cara Allen, Margaux Brewer, Daniel M. Medeiros

**Affiliations:** 1 Department of Ecology and Evolutionary Biology, University of Colorado, Boulder, CO 80309 USA; 2 Department of Zoology, Comenius University in Bratislava, Bratislava, 84215 Slovakia

**Keywords:** Evo-devo, Jaw, Development, Cartilage, Fibroblast, Co-option

## Abstract

The vertebrate head skeleton has evolved a myriad of forms since their divergence from invertebrate chordates. The connection between novel gene expression and cell types is therefore of importance in this process. The transformation of the jawed vertebrate (gnathostome) head skeleton from oral cirri to jointed jaw elements required a diversity of cartilages as well as changes in the patterning of these tissues. Although lampreys are a sister clade to gnathostomes, they display skeletal diversity with distinct gene expression and histologies, a useful model for addressing joint evolution. Specifically, the lamprey tissue known as mucocartilage has noted similarities with the jointed elements of the mandibular arch in jawed vertebrates. We thus asked whether the cells in lamprey mucocartilage and gnathostome joint tissue could be considered homologous. To do this, we characterized new genes that are involved in gnathostome joint formation and characterized the histochemical properties of lamprey skeletal types. We find that most of these genes are minimally found in mucocartilage and are likely later innovations, but we do identify new activity for *gdf5/6/7b* in both hyaline and mucocartilage, supporting its role as a chondrogenic regulator. Contrary to previous works, our histological assays do not find any perichondrial fibroblasts surrounding mucocartilage, suggesting that mucocartilage is non-skeletogenic tissue that is partially chondrified. Interestingly, we also identify new histochemical features of the lamprey otic capsule that diverge from normal hyaline. Paired with our new insights into lamprey mucocartilage, we propose a broader framework for skeletal evolution in which an ancestral *soxD/E* and *gdf5/6/7* network directs mesenchyme along a spectrum of cartilage-like features.

**Supplementary Information:**

The online version contains supplementary material available at 10.1186/s13227-023-00211-1.

## Introduction

The evolution of vertebrates from invertebrate chordates involved a combination of morphological and genomic changes. Vertebrates collectively underwent one round of whole-genome duplication, and both extant jawed and jawless taxa (gnathostomes and cyclostomes, respectively) are thought to have experienced lineage-specific duplications as well [43, 44, 58]. Although invertebrate chordates like amphioxus have a head made partially of cellular cartilage [18], the skeletal system has been greatly elaborated in vertebrates in the form of the skull, jaw, pharynx, fin, and limbs across taxa. In particular, the evolution of the jaw is thought to have facilitated the diversification of vertebrates by allowing a greater range of feeding styles [15]. The origins of the vertebrate jaw are still enigmatic, so the role of novel or co-opted genes in its development is an important part of understanding this process.

The vertebrate jaw is characterized by dorso-ventral patterning in the first pharyngeal arch via nested *dlx* expression and *jagged*-*edn*-*bmp* signaling [54, 60], producing an intermediate domain that expresses a suite of genes like *gdf5* and *nkx3.2* where the future jaw joint [30, 55]. It has been shown that the transcription factor *barx1* is involved with positioning this future jaw joint, as its expression is anti-correlated with this tissue, and its knockdown results in ectopic joint tissue in zebrafish [32, 47, 48]. Conversely, the transcription factor *trps1* is highly correlated with joint tissue and is believed to maintain articular cartilage, with its knockdown resulting in increased hypertrophy [29, 34, 50, 65]. Another important process in articular cartilage formation is TGF-β signaling, with TGFβr2 involved in maintaining the future joint interzone [6, 42, 46, 63]. The roles of *iroquois* proteins in joint formation are still poorly understood, but it has been demonstrated that *irx1, irx5,* and *irx7* have distinct roles in inhibiting chondrocyte maturation and thus some role in joint formation [3, 14]. While joint tissue shares similar ECM expression to other chondrogenic tissues [10, 11], it is surrounded by the viscous liquid of the synovial cavity which contains *lubricin*/*prg4*, a glycoprotein with important roles in joint homeostasis [4, 21, 52]. Despite these advances in our knowledge of gnathostome chondrogenesis and joint formation, we still know little about the evolutionary processes by which these genes were co-opted into the chondrogenic program.

**Fig. 1:**
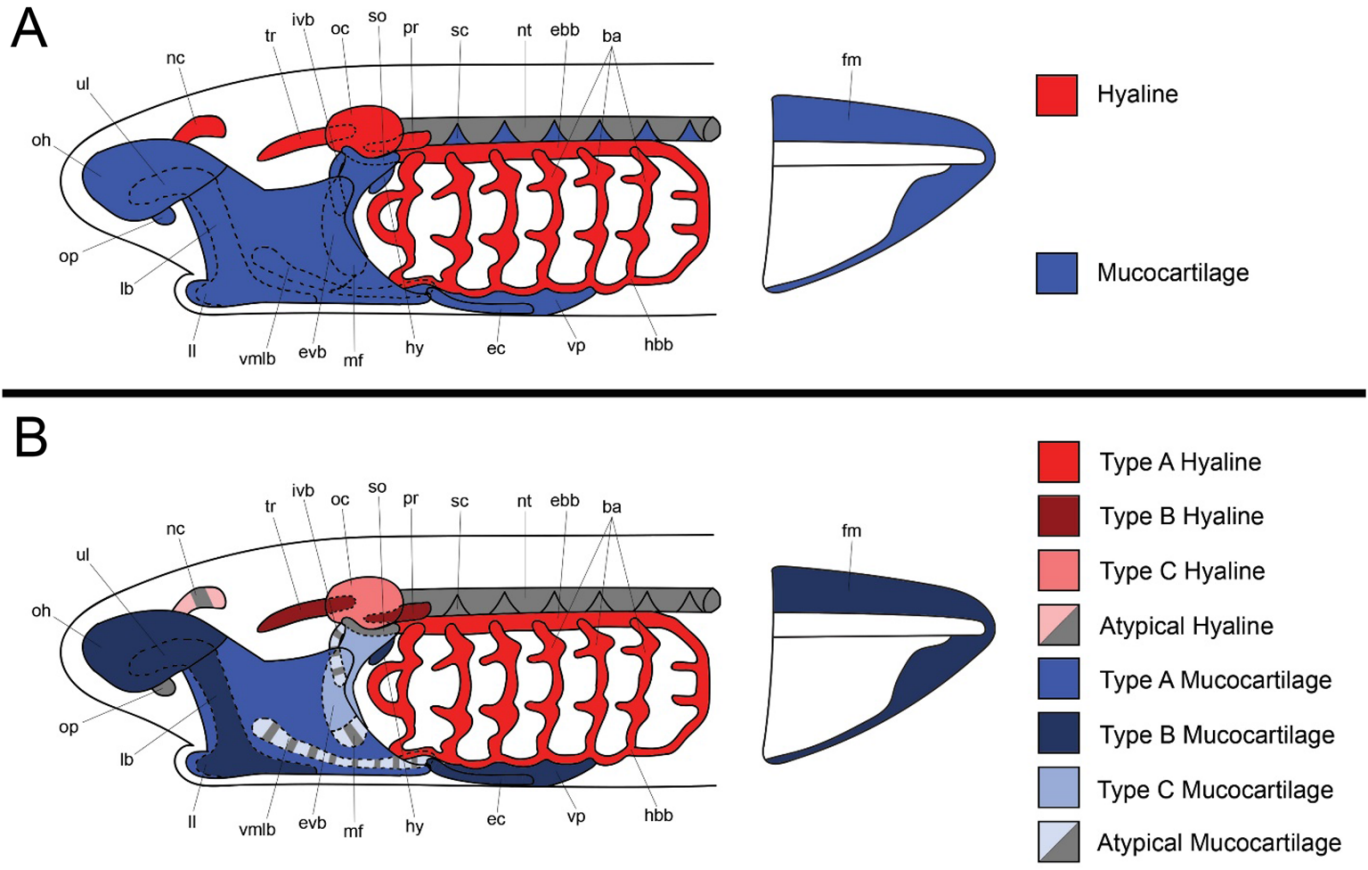
Skeletal anatomy of lamprey ammocoetes. (**A**) Traditional assessment of lamprey skeletal tissues. A firm distinction is present between hyaline-like cartilages (red) and mucocartilages (blue). (**B**) Modified assessment of lamprey skeletal tissues which corroborates previous findings as well as results from this study. The sclerotome and oral papillae have not been as thoroughly studied as other skeletal tissues, so their classification is beyond the scope of this study. For these results, we posit that the absence of type II collagen in the branchial arches in Petromyzon marinus is a derived rather than ancestral trait for lamprey. The distinction between hyaline and mucocartilage is first and foremost determined by histological features, and subtypes are determined by differences in gene expression which are further elaborated in Figure S1. Keywords: ba: branchial arches; ebb: epibranchial bar; ec: endostilic cartilage; evb: external velar bar, hbb; hypobranchial bar; hy: hyoid; ivb: internal velar bar; lb: lateral bar; ll: lower lip; mf: medial flap; nc: nasal capsule; nt: notochord; oc: otic capsule; oh: oral hood; op: oral papilla; pr: parachordal process; sc: sclerotome; so: subotic mucocartilage; tr: trabecular process; ul: upper lip; vlp: ventrolateral plate; vmlb: ventromedial longitudinal bar; vp: ventral pharynx

The jawless lamprey has become an important model organism in our understanding of vertebrate evolution and what skeletal traits may have been present in the common ancestor of cyclostomes and gnathostomes. Despite the differences in morphology between these lineages, both groups shared nested *dlx* expression and *edn* signaling within the pharyngeal arches as well as an absence of *hox* expression in the mandibular arch [9, 22, 53]. These together would mean that much of the patterning of the head skeleton is governed by similar mechanisms. Lamprey furthermore have a diversity of skeletal types throughout their body during development,they have gnathostome-like hyaline in the branchial arches that express master chondrogenic genes like *soxD* and *soxE* homologs [24, 35, 56], and they also have mucocartilage, a connective tissue interspersed throughout the anterior larval head skeleton and fin (Fig. 1A). This tissue has puzzled evolutionary biologists for more than a century, as its morphology and histology are different than gnathostome cartilages and even from lamprey branchial cartilage [38]. Despite these differences, it expresses a suite of similar genes in common with gnathostome joint tissue including *gdf5* [9], lecticans [39], and *col2a1*/*col11a1* homologs among others [37]. These genes are not universally expressed across mucocartilage, and a diversity of subtypes of mucocartilage has been previously noted [8]. While these data imply a relationship between mucocartilage and joint tissue, it is unclear whether these cell types could be considered “biologically homologous” [59]. The relationship between these skeletal tissues has important implications for the Cooption Hypothesis of jaw evolution, which posits that changes in dorsoventral patterning allowed for the recruitment of a mucocartilage-like joint tissue into the mandibular arch intermediate zone [9].

To better understand the relationship between lamprey mucocartilage and gnathostome joint tissue, we characterized expression of lamprey homologs of the cartilage developmental regulators *gdf5*, *barx1*, *trps1*, *tgfβr2,* and *irx1,5,7* as well as the extracellular matrix genes *prg4* and *col9a1* throughout early skeletal development in lamprey Tahara stages 26–28 [51] (for a full glossary of lamprey skeletal terms and abbreviations, see Additional file 2: Table S1). We found that several of these regulator genes and their respective homologs were not expressed in mucocartilage at any developmental time point while others were only temporarily present. This implies that much of the developmental toolkit for cartilage development was only acquired after the divergence of gnathostomes and cyclostomes. We also compared the histochemical affinities of mucocartilage with multiple staining methods. We found that, despite the differences in gene expression between mucocartilage populations, we can only distinguish two subtypes using these musculoskeletal staining techniques. We did reveal histochemical differences in the cartilage of the otic capsule, having features that diverge from traditional gnathostome hyaline and are more akin to elastic cartilage. Taken together, our results suggest that lamprey mucocartilage is not homologous to gnathostome joint tissue but is still governed by a similar core set of chondrogenic factors, meaning that these fibroblast tissues are partially using a shared cartilage gene regulatory network. Paired with our insights into the lamprey otic cartilage, this would mean that cartilage diversity and thus modularity in gene expression were likely present in the last common ancestor of vertebrates, an important step in the later evolution and acquisition of jaws.

## Results

### Expression of *prg4* and *col9a1*

Previous work with lecticans and fibrillar collagens in lamprey have revealed that these genes are spatially distributed throughout connective tissues such that there is no set of these genes that is uniquely specific to lamprey cartilages [37, 39]. We were therefore interested in identifying minor ECM components that may be associated with these cell types, predicting that these genes would behave similarly. Type IX collagen is a FACIT collagen that is well established as a cartilage ECM protein that stabilizes other components like lecticans and fibrillar collagens [5, 12, 57], so we decided to characterize the expression of *col9a1* as well as the aforementioned *prg4* in major lamprey skeletal structures (Fig 1A).

**Fig. 2:**
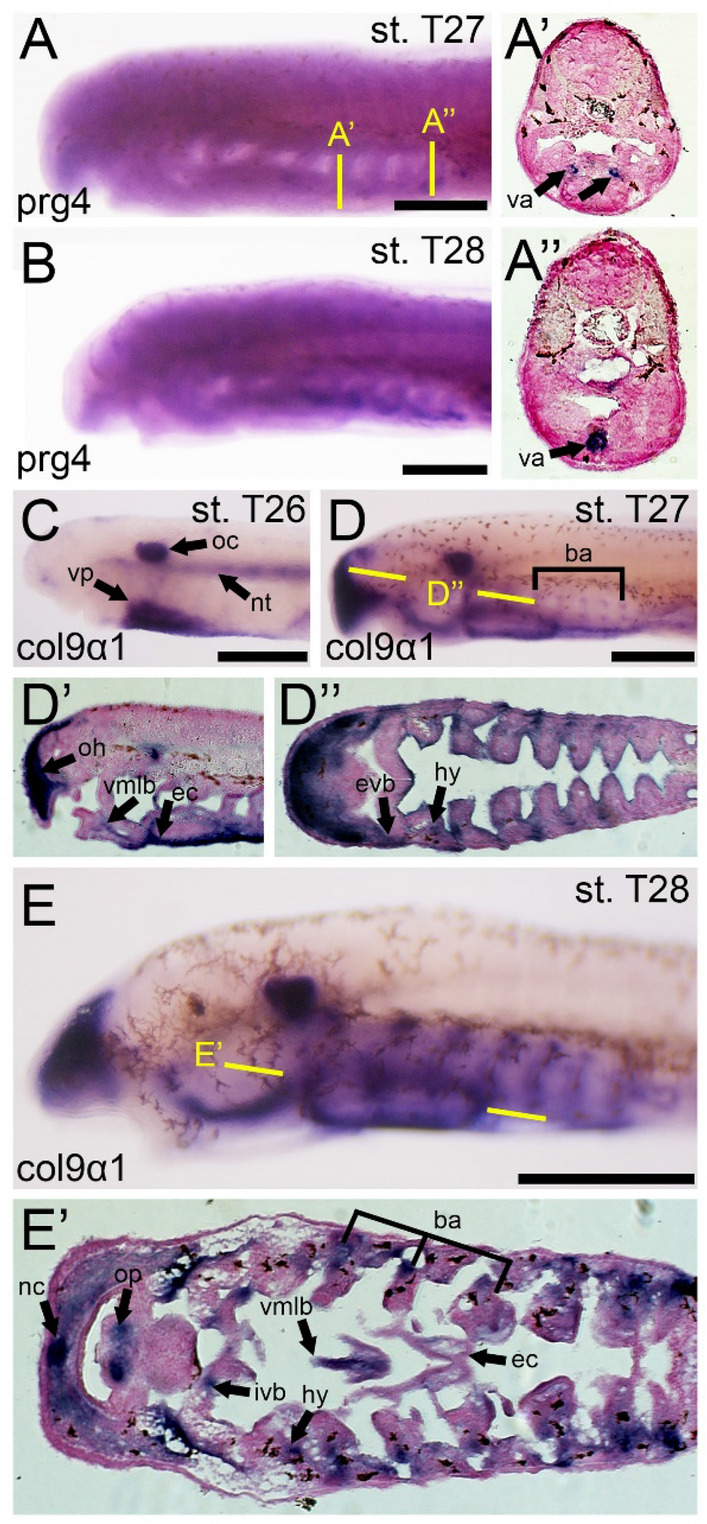
Expression of prg4 and col9a1 in larval lamprey. All scale bars are approximately 250μm. (**A**,**B**) prg4 is detected in the ventral aorta, but no activity outside of this tissue is observed. (**C**) col9a1 is found in the ventral pharynx, otic capsule, and the notochord. (**D**) At stage T27, we observe col9a1 in the oral hood, dorsal and ventral edges of the branchial arches, the hyoid, ventromedial longitudinal bar, and the endostilic cartilage. (**E**) col9a1 is visualized throughout the branchial arches, the ventromedial longitudinal bar, the endostilic cartilage, the hyoid, the internal velar bar, the nasal capsule, and the oral papillae. Keywords: ba: branchial arches; ec: endostilic cartilage; hy: hyoid; ivb: internal velar bar; nc: nasal capsule; nt: notochord; oc: otic capsule; oh: oral hood; op: oral papilla, va: ventral aorta; vmlb: ventromedial longitudinal bar; vp: ventral pharynx

We did not detect *prg4* transcripts until stage T27, and this activity is confined to a small patch along the ventromedial plane (Fig. 2A). Upon sectioning and further review, this expression corresponds to the anterior streams of the ventral aorta. This expression continues through T28 until T29 when it abrogates and expression is no longer visible (Fig. 2B). Based on these findings, we conclude that *prg4* is not associated with any skeletal development in lamprey, meaning that its deployment in skeletogenesis likely arose after gnathostomes and cyclostomes diverged. However, it also remains plausible *prg4* expression in lamprey is a derived condition and have thus secondarily lost expression in the skeletal system.

Conversely, we find abundant *col9a1* activity in lamprey cartilage. When early skeletogenesis begins in lamprey around stage T26, *col9a1* can be visualized in the ventral pharynx, notochord, and otic capsule, with weak expression observed in the nascent pharyngeal arches (Fig. 2C). By stage 27, *col9a1* is found throughout the anterior oral region, otic capsule, and the dorsal and ventral borders of the branchial arches (Fig. 2D). By this stage, the ambiguous expression across the ventral pharynx is becoming specified anteriorly in the ventromedial longitudinal bar (vmlb) and the mucocartilages surrounding the endostyle. These expression domains are almost identical in stage T28, with less expression in the endostylic cartilages being noted (Fig. 2E). It is at this stage that *col9a1* expression in the anterior oral region also presages the cartilage of the nasal capsule, and we begin to see weak expression in the epibranchial and hypobranchial bars. The specification of the ventral pharynx is nearly complete by this stage, and we see *col9a1* activity in this region confined to the most posterior portion. When compared with previous ECM genes in lamprey skeleton, *col9a1* is a fairly reliable marker of traditional hyaline cartilages as well as mucocartilages, with the only *col9a1*-negative tissues of these being the trabecular and parachordal processes (hyaline) and the ventrolateral plate (mucocartilage).

Our findings for *col9a1* and *prg4* are in contrast with previous studies of cartilage ECM genes in that they show much less heterogeneity. *prg4* is absent from all hyaline and mucocartilage at all developmental stages observed, an interesting finding in the larger context of cartilage evolution. Considering the lack of articulation in the lamprey skeleton, we must consider the larger functional roles of this gene. *prg4* is important not only in gnathostome joint cartilage, but also in the surrounding fibroblasts in the synovium, providing structural support as well as signaling to local macrophages [1, 41]. It is thus possible that *prg4* was co-opted in gnathostome joint evolution from the vascular or immune system, its molecular properties beneficial specifically to articulated joints, although this would need to be further explored. In comparison, *col9a1* is among the most specific markers for both hyaline and mucocartilage found to date. As a minor component of the chondrocyte ECM, it is a surprise that its expression is more common in skeletal populations than major components like *col2a1* or *col11a1*. We believe that this may be partially due to lineage-specific changes in *P. marinus*, as it has been demonstrated that the arctic lamprey *Lethenteron camtschaticum* maintains type II collagen in its branchial arches during the onset of chondrogenesis [35], meaning that *col9a1* is likely an accurate marker of skeletogenic mesenchyme despite the changes that have happened in *P. marinus*. Taken together, our results improve our understanding of the ancestral vertebrate cartilage ECM by providing clearer examples of its development, *col9a1* was an integral part of the ancestral chondrocyte ECM, likely in conjunction with type II and XI collagen, while *prg4* was almost certainly co-opted later in evolution.

### Expression of chondrogenic regulatory genes

**Fig. 3:**
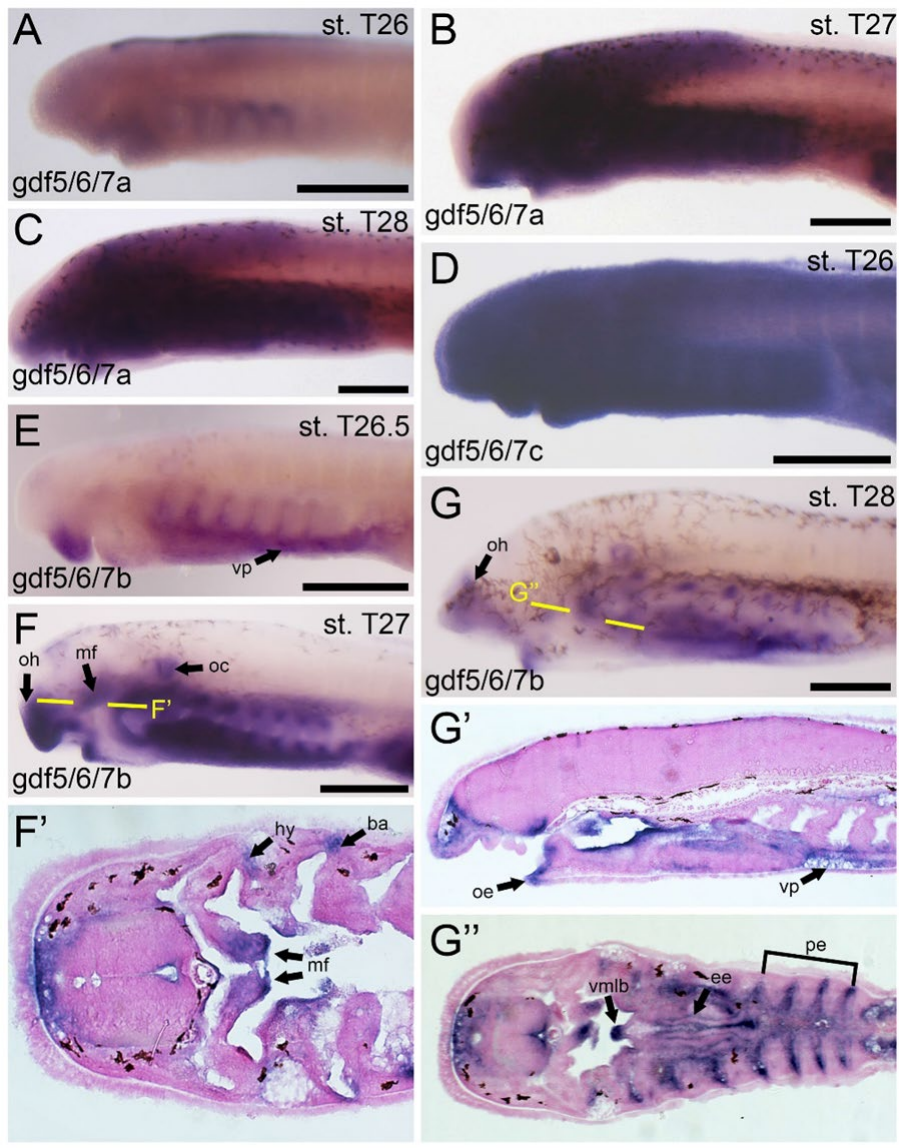
Expression of gdf5/6/7 homologs in larval lamprey. All scale bars are approximately 250μm. (**A**) gdf5/6/7a is identified as a single streak along the dorsal-most part of the body. (**B**,**C**) gdf5/6/7a is ubiquitous throughout the developing head at stages T27 and T28, with no association with any particular tissue. (**D**) gdf5/6/7c is ubiquitous at all stages of interest. (**E**) gdf5/6/7b is observed in the ventral pharynx and in the mesenchyme of the upper lip. (**F**) gdf5/6/7b can be found in the oral hood, medial flap, otic capsule, the dorsal and ventral edges of the branchial arches, and the hyoid. (**G**) gdf5/6/7b is identified in the oral hood, oral ectoderm, ventral pharynx, pharyngeal endoderm, endostilic epithelium, and the ventromedial longitudinal bar. Keywords: ba: branchial arches; ee: endostilic epithelium; hy: hyoid; oe: oral ectoderm; oc: otic capsule; oh: oral hood; mf: medial flap; pe: pharyngeal endoderm; vmlb: ventromedial longitudinal bar; vp: ventral pharynx

Lamprey have three pro-orthologs of the gnathostome *gdf5*, *gdf6*, and *gdf7* genes, known collectively as *gdf5/6/7a,b,* and a newly discovered *gdf5/6/7c*. Of these, *gdf5/6/7b* has been the most thoroughly investigated, with previous data corresponding to this gene [9]. We designed riboprobes for all three genes, having expanded on previous findings for *gdf5/6/7b*. Expression of *gdf5/6/7a* is minimal at Tahara stage 26, having observed a small band running medially along the dorsal top of the body as well as some expression in the presumptive oral endoderm (Fig. 3A). Transcripts are additionally visible in the heart by stages T27 and T28, although expression throughout the head and pharynx is ubiquitous (Fig. 3B, C). Likewise, *gdf5/6/7c* is indeterminate throughout the head and pharynx at all stages examined, with no particular association with any tissue (Fig. 3D). In contrast, we see more specific expression of *gdf5/6/7b* in the skeletal system at these stages. We detect transcripts of *gdf5/6/7b* at stage T26 throughout the mesenchyme of the upper lip and pharynx as well as the ventral endoderm in this region (Fig. 3E). By stage 27, we observe additional expression in the endoderm of the pharynx, the anterior oral region, the medial flap, and expression in the ventral pharynx reveals activity in the epithelium of the endostyle (Fig. 3F). Expression at T28 largely mirrors that seen at stage T27, but direct expression in the dorsal and ventral poles of the branchial arches is also observed at this stage (Fig. 3G). While there is little correlation between *gdf5/6/7a* and *gdf5/6/7c* with the lamprey skeleton, our new *gdf5/6/7b* probe has greatly improved our understanding of its expression, showing new activity throughout the developing skeleton and likely affecting both hyaline and mucocartilage. While previous studies linked its expression to mucocartilage exclusively, *gdf5/6/7b* likely has a role in lamprey chondrogenesis more broadly.

**Fig. 4:**
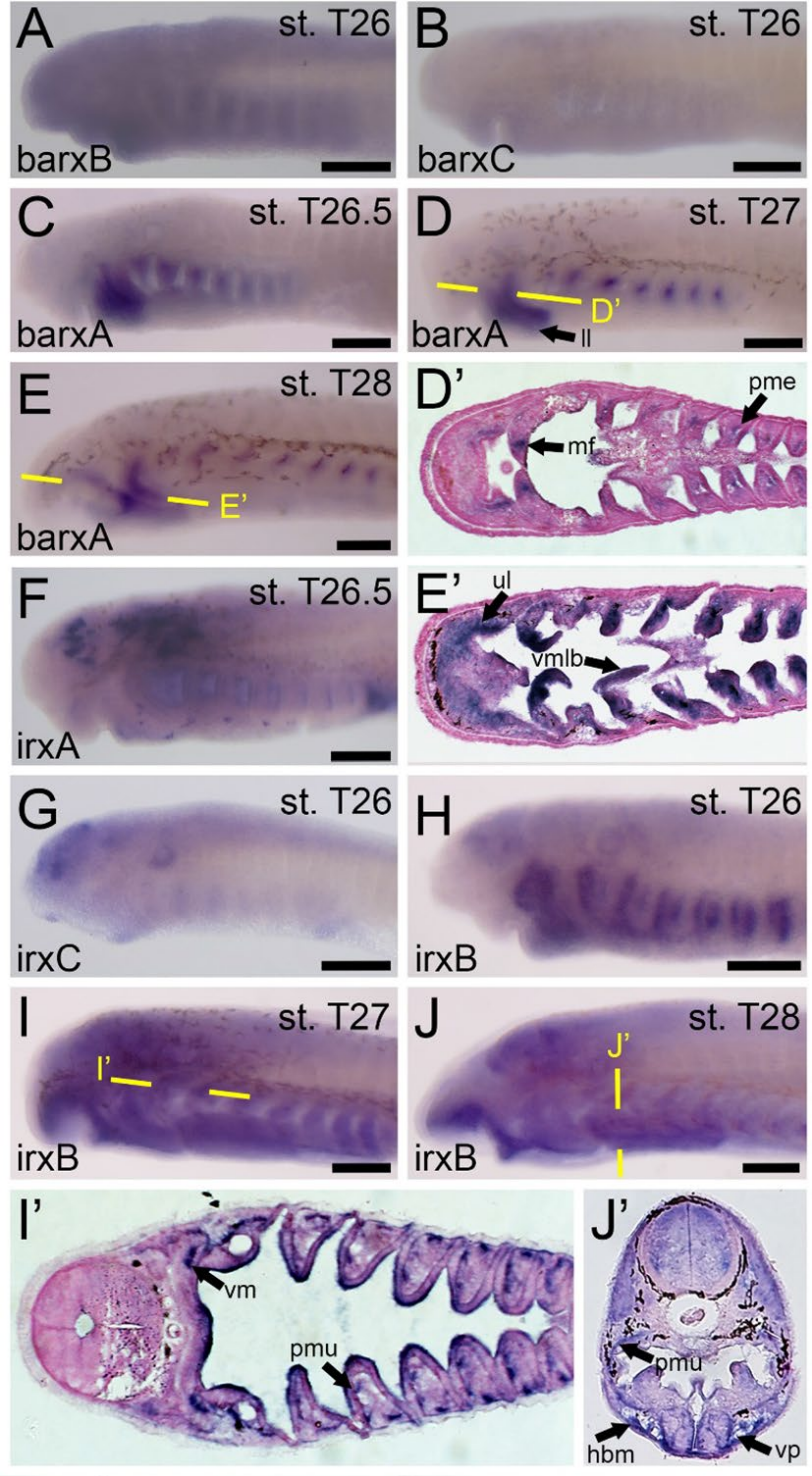
Expression of barx and irx homologs in larval lamprey. All scale bars are approximately 250μm. (**A**,**B**) barxB and barxC homologs are minimally observed throughout all developmental stages of interest. (**C**) barxA is found in the developing branchial arches as well as the velum and lips. (**D**) barxA can be identified specifically in the pharyngeal mesoderm in the branchial arches, the upper and lower lip, and the medial flap of the velum. (**E**) barxA is observed in the upper and lower lips, the ventromedial longitudinal bar, and throughout the non-skeletogenic portion of the branchial arches. (**F**) irxA can be detected in migratory cells throughout the head and pharynx. (**G**) irxC is found specifically in the anterior-most portions of the neural ectoderm. (**H**) irxB is observed throughout the pharyngeal arches as well as portions of the neural ectoderm. (**I**) irxB is found in the musculature of the pharynx and external velar bar. (**J**) irxB can be additionally found in the hypobranchial musculature as well as the ventral pharynx. Keywords: hbm: hypobranchial musculature; ll: lower lip; mf: medial flap; pme: pharyngeal mesoderm; pmu: pharyngeal musculature; ul: upper lip; vm: velar musculature; vmlb: ventromedial longitudinal bar; vp: ventral pharynx

We were next interested in the potential antagonistic relationship between *barx1* and mucocartilage, so we analyzed the expression of *barx* homologs during the aforementioned stages. Previous work has characterized a lamprey *barx* gene [8, 9], but we have identified two additional *barx* homologs of interest. We therefore designed three riboprobes to test the expression of all three paralogs in tandem. Our new probe for the previously studied *barx*, henceforth known as *barxA*, corroborates previous findings for this gene, being expressed at stage T26, T27, and T28 in the medial cranial neural crest cells (CNCCs) of the pharynx and the mesenchyme of the lower lip (Fig. 4C–E). At later stages, this expression specifies to pharyngeal arch derivatives, as expression is even observed in the vmlb (Fig. 4E). In contrast to *barxA*, we see little specific activity in the other *barx* genes. *barxB* expression is weakly expressed throughout the head ectoderm at stage 26 (Fig. 4A), but we identify transcripts in the presumptive CNCCs in the pharynx by T27 and T28 albeit highly unspecific [data not shown]. Likewise, *barxC* expression is weak throughout all stages observed, with only minor activity in the facial ectoderm (Fig. 4B). We find no anticorrelation between *barx* genes and mucocartilage genes, confirming previous findings about these genes. Paired with previous findings on *barx* in gnathostomes, the function of *barx* in ancestral vertebrates was most likely the patterning of pharyngeal arch mesenchyme generally, only later acquiring a more specific role in the patterning of the jaw.

Because *irx7* is a teleost-specific duplicate, we focused on the homologs *irx1* and *irx5* for our study, as these genes are most similar to *irx7 *[3, 14]. Our transcriptomic analyses reveal that lamprey only have three *irx* homologs, named *irxA*, *irxB*, and *irxC*, so we opted to characterize the expression for all three lamprey genes. The expression of *irxA* is mostly confined to earlier stages before this study, being almost absent by stage 27, but we do observe expression at stage T26 in presumptive migratory cells throughout the head and pharynx, with noted activity in the heart as well (Fig. 4F). *irxC* does not appear to be associated with any musculoskeletal tissues, as we detect transcripts of *irxC* throughout the facial ectoderm at all observed stages albeit at lower levels (Fig. 4G). Of the three *irx* genes, *irxB* showed the most relevant activity in the pharynx. We detect transcripts of *irxB* at stage 26 throughout the pharyngeal arches (Fig. 4H). However, *irxB* is localized specifically in the pharyngeal musculature as confirmed by sectioning by stage T27, its expression overlapping much of the expression of the muscle actin gene *ma2* [28, 69] (Fig. 4I). *irxB* expression is more dynamic at stage 28, being identified throughout the pharyngeal mesoderm as well as the medial velum and the anterior oral region (Fig. 4J). Upon sectioning and further review, this expression corresponds to the musculature of the pharynx and hypobranchial process as well as the ventral pharynx. Taken together, our findings suggest that *irx* genes do not have a significant role in lamprey skeletogenesis and were co-opted into skeletogenesis later in vertebrate evolution, their primary role being likely myogenic and neurogenic in nature.

**Fig. 5:**
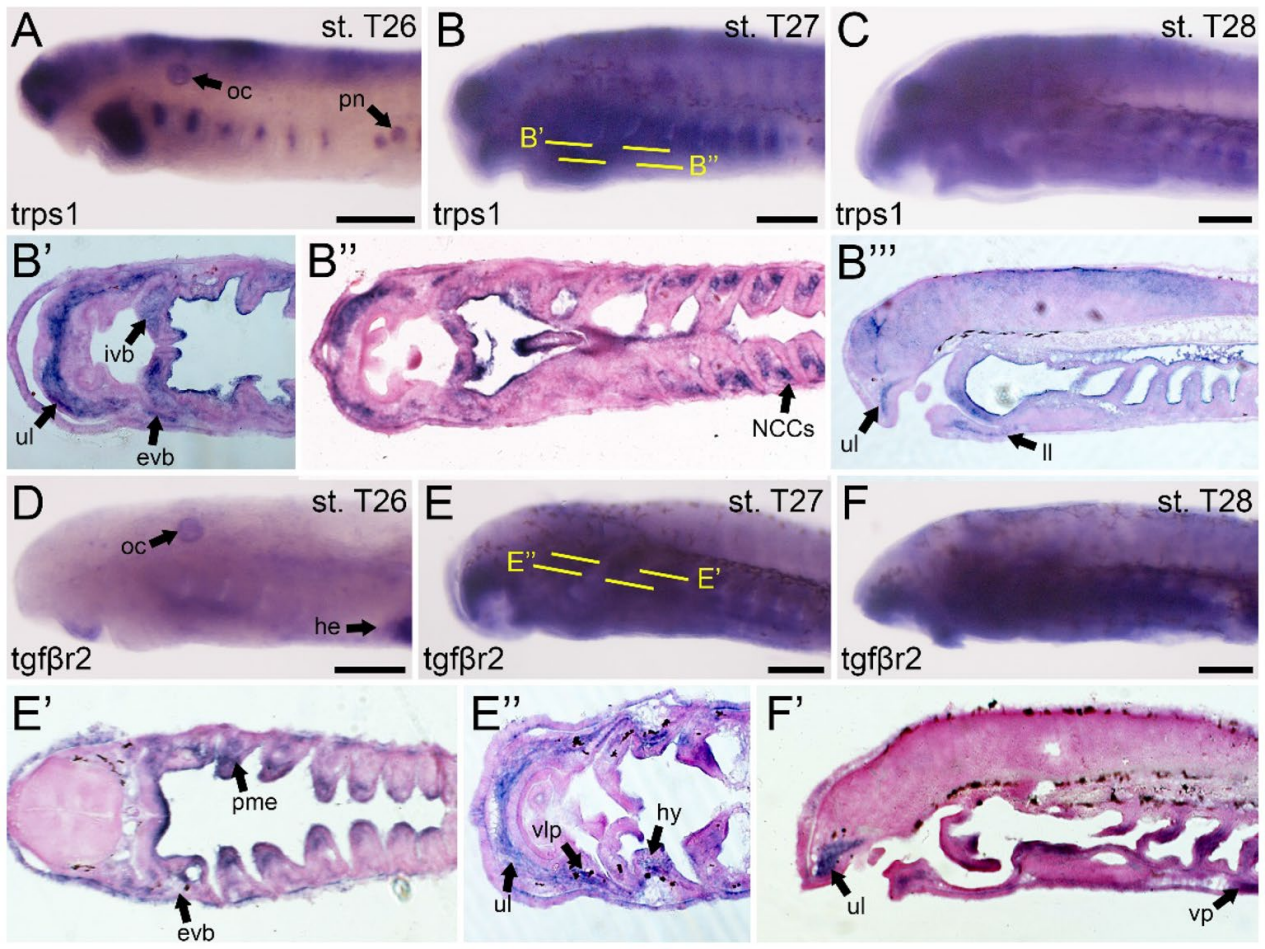
Expression of trps1 and tgfβr2 in larval lamprey. All scale bars are approximately 250μm. (**A**) trps1 can be observed in the pharyngeal arches, neural ectoderm, the otic capsule, and the developing pronephros. (**B**) trps1 is identified in both the external and internal velar bars, the upper lip and lower lip, and the non-skeletogenic neural crest cells in the pharynx. (**C**) trps1 is found in similar locations to those seen in stage T27, although expression in the pharynx retreats along the posterior axis. (**D**) tgfβr2 can be found in the otic capsule, the developing heart, and portions of the upper lip mesenchyme. (**E**) tgfβr2 is observed in the external velar bar, the pharyngeal mesoderm, the upper and lower lip, the ventrolateral plate, and the hyoid. (**F**) tgfβr2 is identified in the same locations at T28 as can be found at stage T27, although expression in the ventral pharynx is more visible at this stage. Keywords; evb: external velar bar; he: heart; ivb: internal velar bar; ll: lower lip; NCCs: neural crest cells; oc: otic capsule; pme: pharyngeal mesoderm; pn: pronephros; ul: upper lip; vlp: ventrolateral plate; vp: ventral pharynx

Our transcriptomic analyses identify only one *trps* homolog with high sequence similarity to that seen in gnathostomes, implying that it is a direct ortholog of *trps1*. At stage T26, we identify *trps1* expression in the pharyngeal arches, brain, and along the dorsal part of the body (Fig. 5A). At stage 27, *trps1* is localized in the upper lip musculature, the mesenchyme of the lower lip and velum as well as the CNCCs and mesoderm throughout the pharynx in a pattern similar to *barx1* (Fig. 5B). By T28, *trps1* activity in the velum is confined to the medial region, and most expression in the lower lip region is no longer present (Fig. 5C). We observe waning expression in the pharyngeal mesoderm along the anterior–posterior axis, with transcripts remaining posteriorly. Despite the importance of *trps1* in gnathostome joint tissue, we do not find a similar correlation with the lamprey homolog in mucocartilage. It is thus likely that *trps1* was co-opted from a network similar to *barx1* that was involved with pharyngeal arch patterning.

We identified one *tgf*β*r2* gene with high sequence similarity to gnathostomes, and we focused primarily on this homolog. At stage 26, we find weak *tgf*β*r2* expression in the mesenchyme of the upper lip and strong expression in the heart (Fig. 5D). By T27, *tgf*β*r2* is found throughout the pharyngeal mesoderm, the musculature of the upper lip, and the mesenchyme of the external velum (Fig. 5E). *tgf*β*r2* expression in the velum shifts medially during stage 28, and we notice a similar intensity of expression throughout CNCC derivatives in the pharynx (Fig. 5F). Expression in the anterior oral region has largely abated by this time, however, but we also observe new activity in the mucocartilage of the hyoid. Taken together, *tgf*β*r2* in lamprey has considerably less roles in skeletogenesis than in gnathostomes, especially so throughout mucocartilage. In the broader context of skeletal evolution, *tgf*β*r2* was highly pleiotropic in the common ancestor of vertebrates and was later co-opted into skeletal development specifically.

Our results show that several genes involved in gnathostome joint formation are almost entirely absent from lamprey mucocartilage at most developmental stages. We notice distinct patterns in these genes, however, being either pharyngeal arch dominant like *barx* and *trps1* or more general throughout the body like *irx* homologs as well as *tgf*β*r2*. These results support the idea that jaw evolution evolved through the cooption of gene regulatory modules both within and outside the pharyngeal arches, suggesting a more complex and stepwise acquisition of these associated genes. We have also found new areas of expression for *gdf5/6/7b* in the developing skeletal system, further supporting the role of these genes as important regulators of lamprey cartilage. These findings together imply that, although a *soxD/E* and *gdf5/6/7* almost certainly govern lamprey chondrogenesis, any deeper parts of this network will be harder to determine.

### Histological and histochemical properties of lamprey mucocartilage

**Fig. 6:**
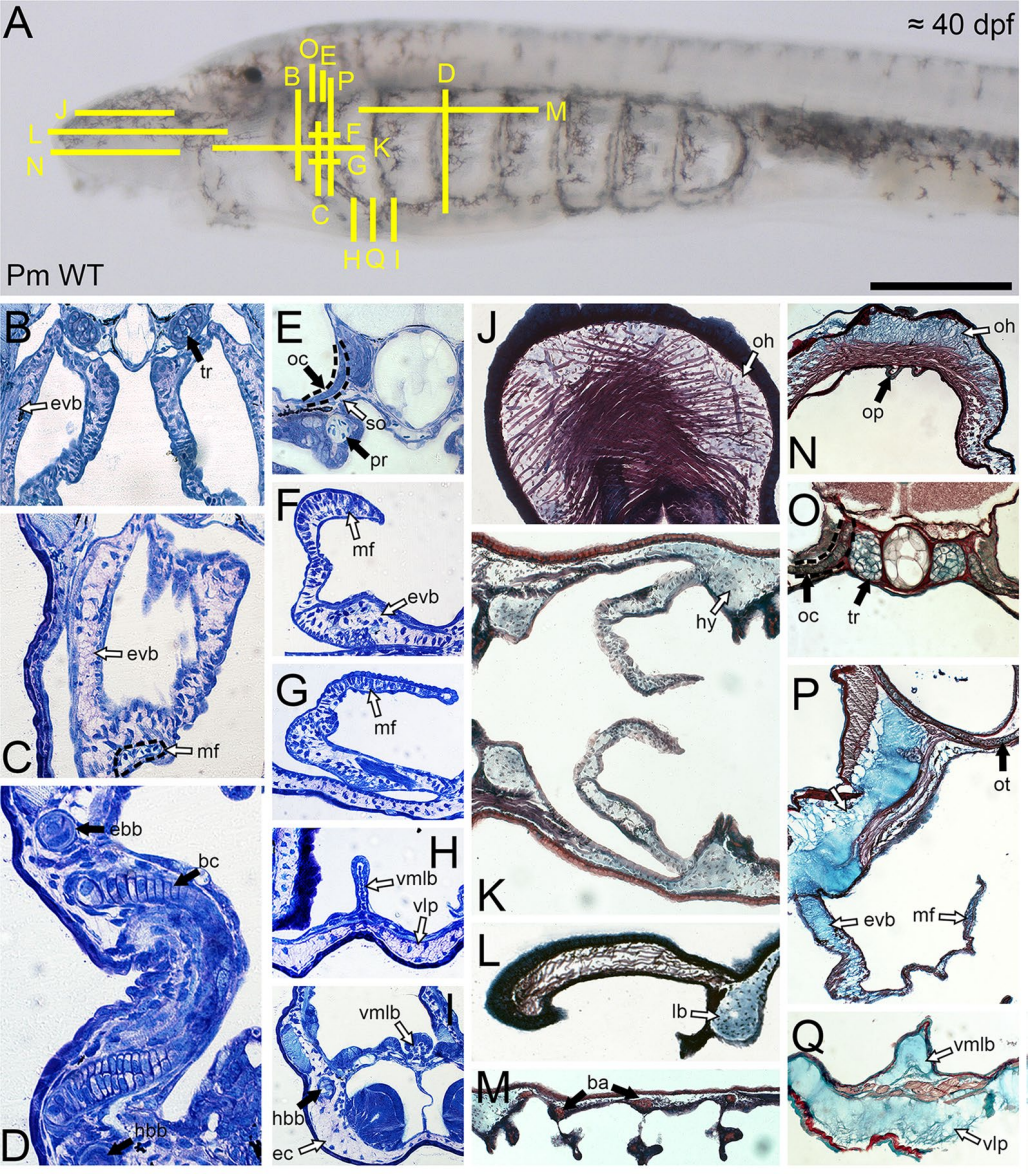
Histology and histochemical properties of lamprey skeletal tissues. Scale bar is approximately 250 μm, and yellow lines indicate the estimated field of view in each panel. Black arrows correspond to hyaline cartilages whereas white arrows indicate mucocartilages. panels B-I are Toluidine Blue staining, panels J-M are Masson Trichrome staining, and panels N-Q are RGB staining. (**A**) Reference image for 40 dpf lamprey larva. (**B**) The tissues of the trabecular processes and the external velar bar contrast the features of hyaline and mucocartilage respectively. (**C**) The external velar bar is compared with features found in the medial flap, indicated by dotted lines. (**D**) The hyaline cartilages of the epibranchial bar, hypobranchial bar, and the branchial cartilages can be observed. (**E**) The hyaline cartilages of the parachordal process and the otic capsule are distinct from one another. (**F**,**G**) Cellular differences are observed between the mucocartilage of the medial flap and the external velar bar. (**H**,**I**) The histology of the ventromedial longitudinal bar is different from that seen in the mucocartilage of the ventrolateral plate and the endostilic cartilage. (**J**) The mucocartilage of the oral hood stands out in contrast to the adjacent connective tissues and muscle fibers. (**K**,**L**) Mucoartilages throughout the pharyngeal reason are largely ubiquitous. (**M**) The staining of the branchial cartilages is distinct from the adjacent mucocartilage. (**N**) Similar to Masson Trichrome, the mucocartilage of the oral hood is distinct from the adjacent dermis and muscle fibers. (**O**) Differences between the hyaline cartilages of the trabecular process and otic capsule are visible under RGB staining. (**P**) Differences between the internal and external components of the velum are minimal using RGB staining. (**Q**) Differences between the ventromedial longitudinal bar and the ventrolateral plate are minimal using RGB staining. Keywords: ba: branchial arches; ebb: epibranchial bar; ec: endostilic cartilage; evb: external velar bar; hbb: hypobranchial bar; hy: hyoid; ivb: internal velar bar; lb: lateral bar; mf: medial flap; oc: otic capsule; oh: oral hood; op: oral papilla; pr: parachordal process; so: subotic mucocartilage; tr: trabecular process; vlp: ventrolateral plate; vmlb: ventromedial longitudinal bar

We first tested whether lamprey hyaline and mucocartilage could be distinguished from one another under multiple histochemical stains. Previous studies have used toluidine blue (TB) as a useful metachromatic stain when viewing musculoskeletal tissues like cartilage [7, 62], so we used TB staining on different lamprey cartilage sections. By Tahara stage 30, both the hyaline cartilage of the trabecles and branchial arches and mucocartilage stain purple, an indication of high polysaccharides, though mucocartilage tends to stain stronger purple (Fig. 6B–D). A notable difference between these skeletal types is that the pericellular matrices of lamprey hyaline are visible using TB staining, with clear demarcation between chondrocyte nests. Additionally, we also note differences between the hyaline cartilage of the trabecles and branchial arches with that seen in the otic capsule, the former having a interterritorial matrix staining strongly blue, a feature more which deviates from normal hyaline (Fig. 6E). We next sought to use the polychromatic stain Masson Trichrome (MT) on paraffin sections of skeletal tissues to validate our findings with TB. By stage T30, mucocartilage is universally indicated by red staining in the chondrocytes, blue staining in the interterritorial matrix, and no visible pericellular matrix between nests (Fig. 6J–L). In contrast, lamprey hyaline cartilage stains strongly red throughout the matrix, but the pericellular matrix does not seem visible (Fig. 6M). These results for mucocartilage and hyaline are largely similar in gnathostomes for skeletogenic and non-skeletogenic connective tissues, respectively. We last tried RGB Trichrome, a recently developed polychromatic series (Picrosirius Red, Fast Green FCF, and Alcian Blue) which has useful applications in distinguishing musculoskeletal cell types from one another including hyaline, elastic cartilage, and fibrocartilage [16]. By stage T30, there are considerable differences in staining between lamprey hyaline and mucocartilage. Hyaline chondrocytes are visible and stain blue, the pericellular matrix is visible and stained red, and perichondrium is visible and likewise stained red (Fig. 6O),in contrast, the interterritorial matrix of mucocartilage stains almost exclusively blue with varying degrees of visible fibers, and cells are weakly visible (Fig. 6N, P, Q). This coincides with the staining affinities of non-skeletogenic connective tissues in gnathostomes [16]. Similar to our TB stains, we see differences in histological staining in the cartilage of the otic capsule compared to normal hyaline with RGB, staining green in a manner similar to that seen in gnathostome elastic cartilage [16] (Fig. 6O). Overall, lamprey hyaline and mucocartilage stain different from one another in both metachromatic and polychromatic tests, the latter cell type more resembling non-skeletogenic connective tissues, but we still notice differences within lamprey hyaline.

We next asked whether we could use the aforementioned stains to distinguish mucocartilages from one another, as their differences in gene expression would suggest that they are different cell types [8]. We were specifically interested in the mucocartilages of the velum, as previous work has suggested that the interior portion of this structure, known as the medial flap, is non-skeletogenic mesenchyme [68]. Compared to TB staining, we find considerably less differences between mucocartilages using polychromatic stains. No discernable differences are detected using MT, and our findings with RGB are likewise minimal. Using TB staining, we were however able to identify some differences. We first looked at the velum, where the interior and exterior halves are clearly separated dorsally by a thin belt of musculature (Fig. 6F). When analyzing the medial flap, it comparatively stains blue and the pericellular matrix between chondrocyte nests is visible (Fig. 6F, G). Proceeding ventrally, most of the medial flap is composed of tightly packed cuboidal cells surrounded by thickened epithelium (Fig. 6G). The posterior mucocartilages can largely be divided between the vmlb, the endostilic cartilages, and the ventral pharynx. The ventral pharynx and endostilic cartilages stain uniformly in a way similar to other mucocartilages, but the vmlb is more similar to the medial flap, with less purple staining and visible pericellular matrices (Fig. 6H, I). Overall, we were only able to identify two distinct mucocartilage types using TB staining, the tissues of the vmlb and medial flap being distinct. The medial flap has been previously considered non-skeletogenic, but we find no evidence that its histological properties should be considered as anything other than mucocartilage, as it shares features with established mucocartilaginous tissues like the vmlb. While further work must be done in larval lamprey to refine MT and RGB methods, our TB stainings show that mucocartilage is histologically more similar than gene expression studies would otherwise imply.

**Fig. 7:**
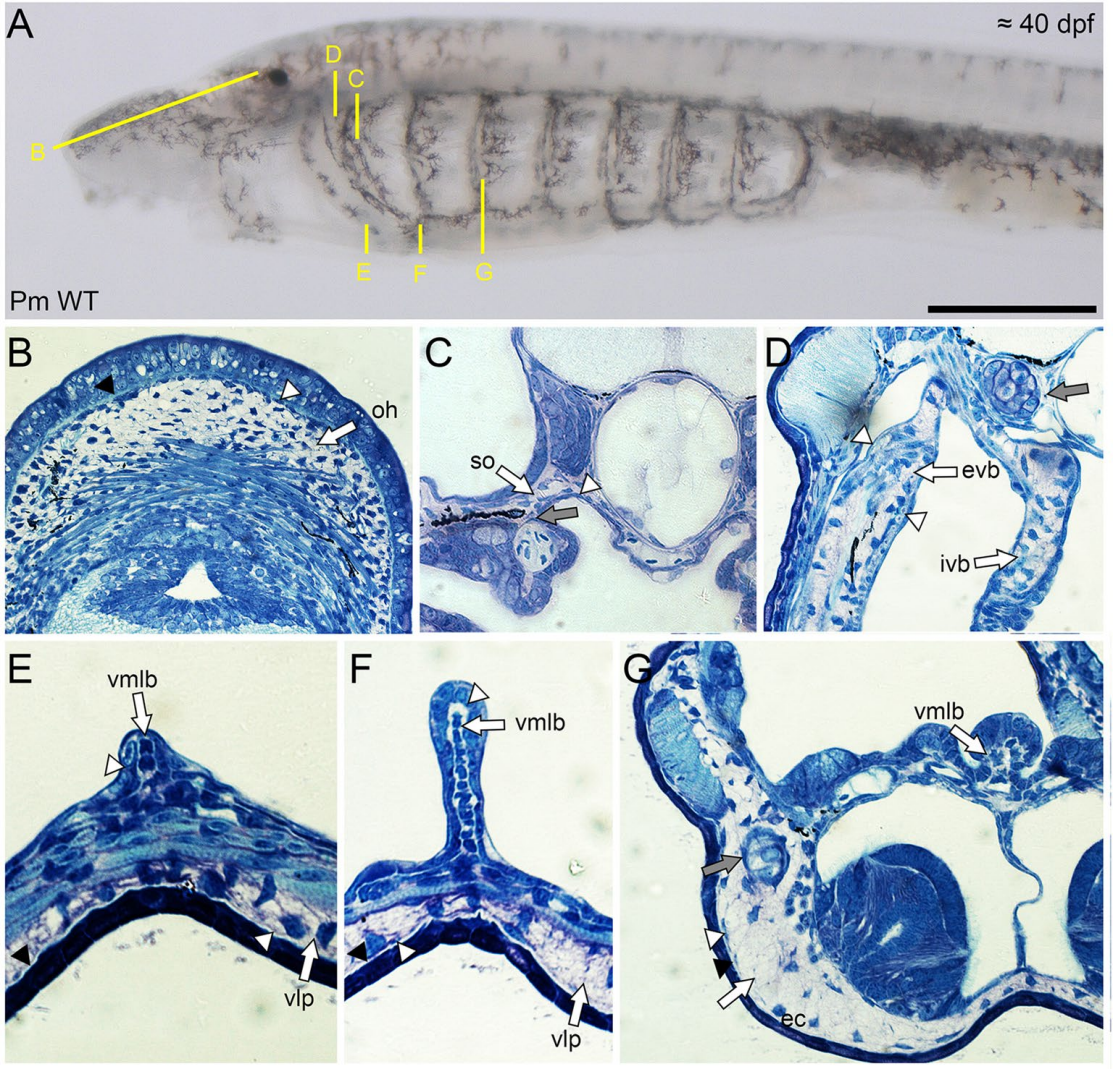
Histology and identification of mucocartilage-adjacent tissues. Scale bar is approximately 250 μm, and yellow lines indicate the estimated field of view in each panel. White arrows correspond to mucocartilage, black arrowheads indicate basement membranes, white arrowheads indicate mucocartilage-adjacent tissues, and gray arrows indicate perichondrium. Panels B-G are all stained with Toluidine Blue. (**A**) Reference image for 40 dpf lamprey larva. (**B**) Mucocartilage of the oral hood. The adjacent cells correspond to the proliferative zone of mesenchyme, but no perichondrium is observed. (**C**) Mucocartilage of the subotic region. Perichondrial tissue can be observed surrounding the parachordal process. The adjacent tissue in mucocartilage is the flattened epithelium of the pharynx. (**D**) Mucocartilage of the internal and external velar bars. Perichondrium is found surrounding the trabecular processThe medial flank of the external velar bar indicates nucleated blood cells while the lateral flank corresponds to flattened epithelial cells. Both sides of the internal velar bar in this panel are surrounded by epithelium. (**E**,**F**,**G**) Mucocartilages of the ventromedial longitudinal bar, endostilic cartilage, and ventrolateral plate. The perichondrium of the hypobranchial bar can be identified in panel G. The presumptive perichondrium of the ventrolateral plate and endostilic cartilage indicate the body wall proper while the that of the ventromedial longitudinal bar corresponds to endothelial projections of the endostyle. Keywords: ec: endostilic cartilage; evb: external velar bar; ivb: internal velar bar; oh: oral hood; so: subotic mucocartilage; vlp: ventrolateral plate; vmlb: ventromedial longitudinal bar

We lastly asked whether we could conclusively identify perichondrial tissues which surround the mucocartilage. Electron microscopy studies reported on perichondrium-like fibroblasts adjacent to the mucocartilage of the ventrolateral plate and ventrolateral longitudinal bar [64], and we looked to further these observations across all mucocartilages. We reasoned that all mucocartilage would have perichondrium encompassing the tissue and that these perichondrial fibroblasts would be largely similar to one another. As a reference, we used TB staining to first identify the perichondrium of cartilage in the trabecular and parachordal processes as well as the branchial arches. With TB staining, the perichondrium stains weakly purple and are stellate in shape, forming a small ring around the chondrocytes (Fig. 7C, D, G). In the ventrolateral plate, we identify the band of cells previously reported to be the perichondrium [64], but these cells stained strongly blue using TB (Fig. 7E–G). Upon further look into the literature, this patch of cells also corresponds to a band of *pax3/7*-positive cells that migrate ventrally from the lateral plate [23, 36], suggesting that these cells are likely part of the ventral body wall rather than perichondrium. We next looked at the vmlb and notice that its perichondrial are more cuboidal in shape than those of the ventrolateral plate (Fig. 7E–G). Moving posteriorly, the reported perichondrial fibroblasts are connected to the epithelium of the endostilic hypobranchial grooves rather than the nearby mucocartilage. Considering that the vmlb itself is surrounded by thickened epithelium that joins posteriorly with the hypobranchial grooves of the endostyle, it is more likely that its “perichondrium” is either connective tissue associated with the pharyngeal epithelium or is itself epithelium. We next tested the mucocartilages that were not in the aforementioned work, and we find that none of them (oral hood, subotic mucocartilage, velum) had perichondrium-like tissues. In the case of the velum, the cells surrounding the mucocartilage are contiguous with the thickened epithelium that can be found at the medial most point of the velum, supporting that these are likely flattened epithelial cells rather than fibroblasts (Fig. 7B–D). Without any unifying characteristics of the reported perichondrium across tissues and its absence in several others, our results together posit that mucocartilage is likely not surrounded by perichondrial tissue. Combined with its broader histological features, this lamprey cell type should be largely considered non-skeletogenic connective tissue.

Our results show that lamprey mucocartilage is more homogenous at the histological level than gene expression assays would predict, and this tissue consistently stains similar to mesenchyme and fibroblasts in gnathostomes. Toluidine blue is able to distinguish the vmlb and the medial flap from other mucocartilages, however, suggesting that there is still some heterogeneity in these lamprey skeletal tissues. We also found differences in lamprey hyaline types, with TB and RGB staining revealing the cartilage of the otic capsule to deviate from the features of traditional hyaline and, in the case of RGB staining, reveal features similar to elastic cartilage. When taking a more holistic view of mucocartilage, we were unable to find any perichondrium surrounding these tissues. Our histochemical assays of larval lamprey cartilage are among the most comprehensive to date, and these comparative methods help give deeper insight into lamprey musculoskeletal anatomy. Paired with our gene expression analyses, these provide a powerful tool to assess the cellular identity of tissues.

## Discussion

In this study, we compared lamprey mucocartilage and hyaline cartilage through more extensive means like comparative histochemistry, and we further looked at differences in gene expression between them. Morphologists have wondered about the homology and origin of lamprey mucocartilage for more than a century [38], and it has only been with renewed interest and an improved understanding of histology, genetics, and evolution that we have been able to address it. We have previously suggested that skeletal tissues exist along a spectrum of connective tissues with chondrocyte-like features [38], and our findings help further elaborate this schema. While mucocartilage is not homologous to gnathostome joint tissue and almost certainly not a skeletal tissue per se, these fibroblasts show a range of skeletal-like properties that indicate levels of chondrification. Paired with our results that show skeletal heterogeneity in lamprey hyaline-like cartilages, specifically the otic capsule, we posit that a combination of chondrocyte–fibroblast interactions helped generate the diversity of skeletal tissues we see in vertebrates, a critical aspect of vertebrate skeletal evolution and specifically the gnathostome jaw.

### Lamprey mucocartilages are partially chondrified fibroblasts

The earliest reports of mucocartilage consistently described this tissue as fibroblast-like, yet still prescribed it cartilage-like features such as a strong responsivity to Alcian blue staining and a ground substance rich in hyaluronic acid [38]. The idea that mucocartilage was surrounded by perichondrial tissue further complicated our understanding of this cartilage-like cell type, supporting that it was a cartilage that significantly deviated from traditional chondrocyte development. Our results here suggest that mucocartilage does not have a perichondrium, supporting histological data that would categorize it as fibroblasts with cartilage properties rather than cartilage with fibroblast properties. Fibroblasts themselves are somewhat unclear in features that distinguish them from other connective tissues, so it will be important to identify these in order to determine what mucocartilage is and the extent that it has skeletal properties.

Despite decades of usage in stem cell biology, fibroblasts still do not have a fixed definition [17, 45]. The conflation of the word itself with other cell types like fibrocytes and mesenchymal stem cells (MSCs) further show that we still do not understand how fibroblasts differentiate and mature nor even if these cells are terminally differentiated. The extent that mucocartilage is differentiated has important implications of lamprey metamorphosis, a period in which this tissue reverts to a mesenchymal state before redifferentiating into traditional cartilage [2]. Although there are similarities in histology and broad gene expression between fibroblasts and MSCs, DNA methylation patterns may prove to be an effective marker to distinguish them, supporting the claim that these cells mostly represent a spectrum of multipotency states [45]. Future work is needed to determine where mucocartilage exists along this continuum and how they are altered during metamorphosis.

The differences in gene expression between mucocartilage and normal fibroblasts is therefore of great interest, as mucocartilage does have Alcian staining affinities closer to cartilage than non-skeletal connective tissues [67]. Paired with differences in expression of key ECM genes involved with connective tissues like *col1a2*, *col2a1*, *col3a1*, and *col9a1* as well as lecticans [37, 39], mucocartilage shares more ECM similarities with cartilage in comparison to other fibroblasts. The differences in expression of these genes between various mucocartilages need to also be considered in the broader context of development, as these differences may reflect subtypes of this tissue or differences in maturity and differentiation. Three key examples of this are the oral hood, the ventral pharynx, and the fin fold, which collectively show diminishing expression of *col1a2a* and *col3a1a* during later development, this expression being progressively confined to the ends, respectively. This would suggest that Type I and III collagen, traditionally markers of fibroblasts and mesenchyme in gnathostomes, may be specific to the proliferative zone of these mucocartilage and thus help resolve some of the heterogeneity that we see across these tissues. In contrast to previous findings, we propose a more simplistic model for lamprey skeletal types, the main criteria distinguishing them being the presence or absence of lecticans, major and minor cartilage ECM collagens like type II and IX, respectively, and core chondrogenic regulators like *soxD/E* homologs and *gdf5/6/7* (Fig. 1B) (Additional file 1: Fig. S1).

### Skeletal modularity is an ancestral feature for vertebrates

Gnathostomes have evolved a diversity of skeletal tissues over the past five hundred million years, using distinct types of cartilage, bone, and a variety of cells with intermediate features between them [13]. The fossil record also shows a range of mineralizing tissues across specimens, implying that skeletal diversity was present in ancient vertebrates as well. Because the phylogenetic position of cyclostomes among fossil and extant vertebrates is still unclear [20, 31], the skeletal repertoire seen in extinct groups like heterostracans and anaspids cannot tell us alone whether this diversity was present in the common ancestor of gnathostomes and cyclostomes. Even though mucocartilage is most likely a derived tissue and therefore not symplesiomorphic for vertebrates, it has several components of a shared cartilage gene regulatory network. Our results here provide a more holistic view of lamprey mucocartilage from the perspective of vertebrate skeletal evolution, and our findings hint that non-skeletal cells like fibroblasts can display chondrocyte-like properties via regulation by *gdf5/6/7* homologs. This would mean that core cartilage regulatory genes can act more broadly across connective tissues, an important step in the diversification of vertebrate skeletal tissues.

**Fig. 8:**
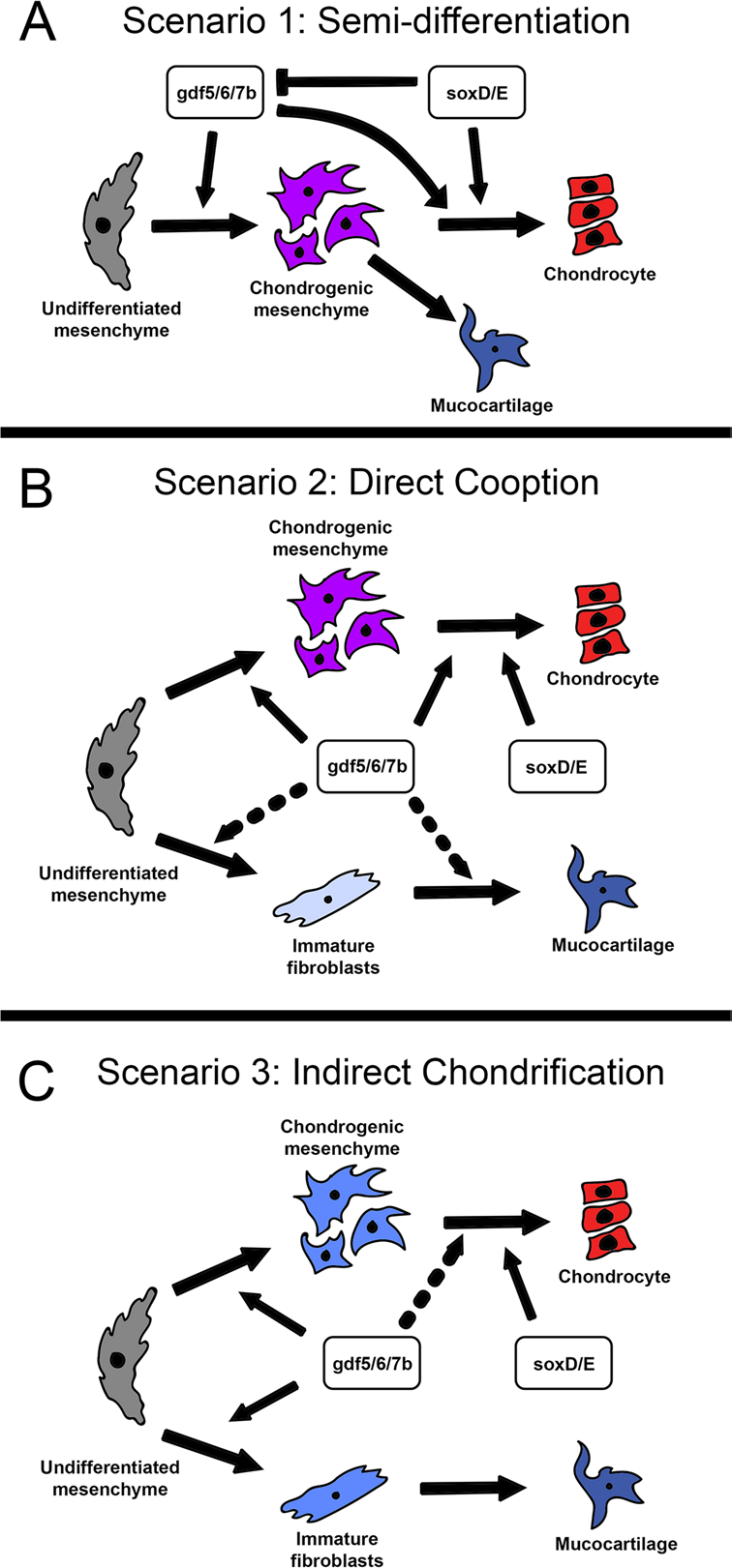
Possible scenarios for the evolution of skeletal modularity in lamprey. For these results, we posit that the absence of type II collagen in the branchial arches in Petromyzon marinus is a derived rather than ancestral trait for lamprey. (**A**) The Semi-Differentiation Hypothesis states that lamprey mucocartilage is the result of halted differentiation wherein a semi-differentiated chondrogenic mesenchyme commits instead to the fibroblast lineage. In this scenario, absence of traditional soxD/E homologs permits the development of mucocartilage, as direct or indirect inhibition of gdf5/6/7 signaling promotes hyaline chondrogenesis. This scenario would partially explain the presence of multiple chondrogenic ECM genes in mucocartilage like type II and IX collagen. (**B**) The Direct Cooption Hypothesis states that the ancestral function of gdf5/6/7 signaling was primarily chondrogenic and was only later coopted in mucocartilage fibroblasts. This scenario would partially explain the diversity of mucocartilage-like phenotypes with respect to hyaline. (**C**) The Indirect Chondrification Hypothesis states that the ancestral gdf5/6/7 module was a generalist pathway involved in mesenchyme differentiation and only later acquired chondrogenic function. This scenario would partially explain the differences in lecticans between these tissues, as one was specific for mesenchyme and the other for developed hyaline

The manner in which *gdf5/6/7* and *soxD/E* homologs drive chondrification across skeletal and non-skeletal cells in lamprey is still unclear. Previous studies which tested *soxD/E* expression in lamprey found that these genes are mostly restricted to hyaline cartilage during skeletogenesis, although *soxE3* has also been detected in the external velum [24, 35]. With the exception of the latter, these tissues also correspond to *lecC*-positive cells [39], suggesting that there is a connection between *soxD*, *soxE1/2*, *lecC*, and traditional hyaline cartilage. Conversely, we see *gdf5/6/7* activity across both hyaline and mucocartilages. We see *lecA* as the dominant lectican across the majority of these mucocartilages, and this expression is even observed in the pre-chondrogenic mesenchyme of the branchial arches earlier in development [39]. To explain the connection between *sox*-*lecC* and *gdf5/6/7*-*lecA* in skeletal development, we posit three scenarios. In the first, the *gdf5/6/7*-*lecA* module is specific to pre-chondrogenic mesenchyme, and the mucocartilage phenotype is due in part to the absence of *soxD* and *soxE1/2* (Fig. 8A). *soxD* and *soxE1/2* would likely downregulate *lecA* and other aspects of *gdf5/6/7* signaling, but *soxE3* evolved new functions that change its interaction with this pathway, considering that the exterior velar bar is a pharyngeal arch mucocartilage yet expresses *soxE3*. In the second scenario, the *gdf5/6/7*-*lecA* module was ancestrally a cartilage module that was later co-opted into fibroblast tissues (Fig. 8B). This scenario would allow us to explain the presence of type II and IX collagen in mucocartilage among others, as these cartilaginous ECM genes were likewise co-opted. In the third scenario, the *gdf5/6/7* module is more generalist in function in mesenchyme during development and only later acquired chondrocyte-like expression of genes like lecticans, fibrillar collagens, and type IX collagen (Fig. 8C). The mechanism through which this happened is unclear, but this scenario allows us to reconcile the pleiotropy we see with *gdf5/6/7* signaling as well as inconsistencies in gene expression across all cell types. Each of these scenarios has different implications for the evolution of skeletal diversity in lamprey. The first scenario would imply that mucocartilage is a pre-chondrogenic mesenchyme that semi-differentiates and commits to the fibroblast lineage later in development, the second would imply that mucocartilages are fibroblasts that directly co-opted cartilage modules, and the third would imply that mucocartilages are fibroblasts that indirectly acquired chondrocyte-like properties. Functional studies will be necessary to determine the exact relationship between these genes and their respective cell fates. While we are still uncertain about the specific changes which created mucocartilage, it remains possible these intermediate cells are the result of interactions in mesenchyme between chondrocyte and fibroblast-associated pathways.

The question remains whether the repertoire of cartilage and cartilage-like tissues found in lamprey were present in the common ancestor of vertebrates. We have previously discussed the similarities between lamprey mucocartilage and hagfish pseudocartilage [38], and it is likely that it is a closely-related cell type among cyclostomes. There are no tissues in gnathostomes yet which have the same myriad of features as mucocartilage/pseudocartilage, implying that either jawed vertebrates lost the cell type or it is cyclostome-specific. Specifically to lamprey, this cell type has important functional roles in forming the oral cavity and providing hydrostatic support during filter feeding [25]. It has been suggested that the common ancestor of vertebrates was a burrowing filter feeder in a way similar to that seen in the invertebrate amphioxus as well as larval lamprey [40], meaning that much of the oropharyngeal anatomy at this stage may be similar. Although the tissue itself might have been structurally different, it is likely that chondrified fibroblasts were present in the common ancestor, being potentially lost in gnathostomes later as the oral cavity changed or developed different histologies from its previous form. Alongside these tissues, we must also consider whether the lamprey otic capsule cartilage is a homolog of gnathostome elastic cartilage. Our findings here posit that the otic capsule has different histological properties than those seen in the hyaline of the branchial arches and trabecles, but these alone are not sufficient for homology. The differences between gnathostome elastic cartilage and hyaline seem minimal, as comparative studies between them only reveal a small set of different genes [66, 70]. Considering that lamprey hyaline is also distinguished by the presence of elastin-like genes like *lamprin* and *pharymprin* [26, 27, 68], it is likely that the staining differences between lamprey otic and hyaline cartilage are due to other differences in ECM structure than those between gnathostome elastic and hyaline cartilage. These two skeletal types are thus different hyaline types and we cannot posit whether this distinction was present in the common ancestor. Taken together, our findings support a complex skeleton at the base of vertebrates, although the specifics of this skeletal diversity and its deployment are still unclear.

Gnathostomes have been the dominant lineage of vertebrates for more than three hundred fifty million years in part to vast morphologies that have diversified land, air, and water. Part of this success has been attributed to the evolution of the jaw, and our results here find no evidence of a joint-like skeletal tissue in lamprey, suggesting that both the jaw joint tissue cells and the dorsoventral patterning required for jaws were a later innovation. While there is no direct lamprey homolog for joint tissue, our results do suggest that the ancestral vertebrate repertoire of cartilage and cartilage-like cells was highly diverse, an important step in the development and evolution of vertebrate morphologies. These different cells likely stem from changes to a core chondrogenic module, whether in the case of otic cartilage as smaller modifications to hyaline cartilage or in the case of mucocartilage as partially chondrified fibroblasts. This diversity of structural tissues was likely critical for the development of not only the gnathostome oral cavity, but also that of cyclostomes and other jawless fishes. Based on our findings, we posit that skeletal modularity was pivotal for the evolution of gnathostomes, traces of which can be detected even in their distant ancestors.

## Methods

### Isolation of lamprey homologs

Lamprey collagen sequences were tiled from transcriptomic reads of Tahara st. 26.5 embryos and adult oral disc tissue that were previously gathered and submitted to GenBank [61]. Sequences from these files were used for our phylogenetic and syntenic analyses. For in situ hybridizations for *barxA*, *trps1*, and *gdf5/6/7b*, primers were designed from lamprey genomic sequence to amplify conserved exon sequences, which were cloned into the pJet1.2 vector. For the remainder of the lamprey genes, 500–550 bp regions from transcriptomic sequences were selected and ordered as fragments in pUC57-amp vector from Synbio Tech^©^.

### Embryo collection and staging

Embryos for in situ hybridization were obtained from adult spawning-phase sea lampreys (Petromyzon marinus) collected from Lake Huron, MI, and kept in chilled holding tanks as previously described [33]. Embryos were staged according to the method of Tahara [51], fixed in MEMFA (Mops buffer, EGTA, MgSO_4_, and formaldehyde), rinsed in Mops buffer, dehydrated into methanol, and stored at − 20 °C.

### In situ hybridization

Riboprobes were made for anti-sense fragments using SP6 RNA Polymerase. Sequences for probes and genes are available upon request. In our experience, full-length *P. marinus* riboprobes, or riboprobes generated against untranslated regions of P. marinus transcripts, give higher background than short riboprobes against coding sequences. We believe that this is because lamprey noncoding sequences, especially 3′ UTRs, often have an excessive GC-repeat content, causing corresponding riboprobes to hybridize nonspecifically to off-targets. To mitigate this, we made short 550-bp riboprobes against coding regions and used a high-stringency hybridization protocol [9, 49]. Key parameters of this protocol include post-hybridization washes at 70 °C and the use of a low-salt, low-pH hybridization buffer (50% formamide, 1.3 × SSC, pH 5.0; 5 mM EDTA, pH 8.0; 50 μg/mL tRNA; 0.2% Tween-20; 0.5% CHAPS; and 100 μg/mL heparin).

### Histology, histochemistry, and sectioning

After in situ hybridization, embryos were postfixed in 4% paraformaldehyde/PBS (4 °C, overnight), rinsed in PBS, cryo-protected with 15% sucrose/PBS, embedded in 15% sucrose, 20% gelatin/PBS (37 °C, overnight), and 20% gelatin/ PBS (37 °C overnight), frozen in liquid nitrogen, and mounted in OCT compound (Miles). Cryo-sections of 14 μm were collected on Super Frost Plus slides (Fisher Scientific), counterstained using Nuclear Fast Red (Vector Laboratories), and dehydrated and mounted in DPX (Fluka) [19]. For Masson Trichrome and RGB staining, formaldehyde-ixed embryos were rinsed in PBS, dehydrated with alcohol and infiltrated with Histoclear II, and lastly embedded in Paraplast^©^ overnight. Sections of 8–10 μm were collected on Super Frost Plus slides (Fisher Scientific). All slides were rehydrated and cleared with Histoclear II for 15 min before staining. For TB staining, embryos were progressively infiltrated in Infiltration Solution (JB4 Monomer A/benzoyl peroxidase)/EtOH for several hours before being left overnight in 100% Infiltration Solution. The following day, embryos were embedded in 25:1 Infiltration Solution and JB4 Monomer B. Sections of 4–6 μm were collected on Super Frost Plus slides (Fisher Scientific) using a glass knife.

Toluidine Blue, Masson Trichrome, and RGB staining were done on cleared slides with minor modifications to each. For Toluidine Blue, dehydrated slides were treated in 0.1% Toluidine Blue at 70 ℃ for 30 s, washed in three series of distilled water for 3 min each, and progressively dehydrated, recleared, and mounted in DPX (Fluka). For Masson Trichrome, slides were treated in Bouin Solution at 56 ℃ for 15 min, Weigert’s Solution (Sigma Aldrich) for 5 min, 1% Biebrich Scarlet/Acid Fuchsin (Sigma Aldrich) for 5 min, 5% PTA/PMA (Sigma Aldrich) for 10 min, 2.5% Aniline Blue (Sigma Aldrich) for 8 min, 1% acetic acid for 1 min, and lastly progressively dehydrated, recleared, and mounted in DPX (Fluka). For RGB staining, slides were treated in 1% Alcian blue pH 2.5 for 20 min, 1% Fast Green FCF for 20 min, 1% Picrosirius for 30 min, two washes in 1% acetic acid for 5 min each, and lastly progressively dehydrated, recleared, and mounted in DPX (Fluka).

### Imaging

Whole-mount in situ hybridized *P. marinus* embryos and larvae were photographed using a Carl Zeiss Axiocam MRc5, Carl ZeissDiscovery V8 dissecting microscope, and Axiovision 4.9.1 software. Sections were photographed using a Carl Zeiss Imager A2 compound microscope.

## Supplementary Information

**Additional file 1: Figure S1**. Differences in gene expression between skeletal subtypes. The main five criteria for gene expression correspond to soxD/E homologs, gdf5/6/7b, lecticans, and type II/IX collagens. Because gdf5/6/7b is a signaling ligand, we consider that either direct expression or proximity to a tissue that expresses it would be functionally similar. “Atypical” is defined as missing a component which all other hyaline or mucocartilages, respectively, would have, prioritizing ECM genes over regulatory genes.

**Additional file 2: Table S1.** Glossary of lamprey skeletal terms used in this paper and their abbreviations.

## Data Availability

The datasets presented in this study are available upon request to the authors.
