# Supplementary material for: Cartilage diversification and modularity drove the evolution of the ancestral vertebrate head skeleton
Source: EvoDevo. 2023 May 5;14:8. doi: 10.1186/s13227-023-00211-1 (PMC10161429; doi:10.1186/s13227-023-00211-1)
Supplement: Supplementary file 1 — Additional file 1: Figure S1. Differences in gene expression between skeletal subtypes. The main five criteria for gene expression correspond to soxD/E homologs, gdf5/6/7b, lecticans, and type II/IX collagens. Because gdf5/6/7b is a signaling ligand, we consider that either direct expression or proximity to a tissue that expresses it would be functionally similar. “Atypical” is defined as missing a component which all other hyaline or mucocartilages, respectively, would have, prioritizing ECM genes over regulatory genes. [file 13227_2023_211_MOESM1_ESM.pdf]

Skeletal Type	soxD/E	gdf5/6/7b	lectican	type II	type IX
Type A Hyaline	✓	✓	C	✓	✓
Type B Hyaline	✓		C	✓	
Type C Hyaline	✓	✓	C, A		✓
Atypical Hyaline		✓	C		✓
Type A Mucocartilage			A		
Type B Mucocartilage		✓	A	✓	✓
Type C Mucocartilage	✓	✓	A	✓	✓
Atypical Mucocartilage		✓			✓
