# Supplementary material for: Cartilage diversification and modularity drove the evolution of the ancestral vertebrate head skeleton
Source: EvoDevo. 2023 May 5;14:8. doi: 10.1186/s13227-023-00211-1 (PMC10161429; doi:10.1186/s13227-023-00211-1)
Supplement: Supplementary file 2 — Additional file 2: Table S1. Glossary of lamprey skeletal terms used in this paper and their abbreviations. [file 13227_2023_211_MOESM2_ESM.pdf]

Table S1. Glossary of lamprey skeletal terms used in this paper and their abbreviations

Abbreviation	Skeletal Term
ba	branchial arches
ebb	epibranchial bars
ec	endostilic cartilage
evb	exterior velar bar
hbb	hypobranchial bars
hy	hyoid
ivb	interior velar bar
lb	lateral bar
ll	lower lip
mf	medial flap
nc	nasal capsule
nt	notochord
oc	otic capsule
oh	oral hood
op	oral papillae
pr	parachordals
sc	sclerotome
so	suborbital cartilage
tr	trabeculae
ul	upper lip
vmlb	ventromedial longitudinal bar
vp	ventral pharynx
